# Microbial Diagnostic Array Workstation (MDAW): a web server for diagnostic array data storage, sharing and analysis

**DOI:** 10.1186/1751-0473-3-14

**Published:** 2008-09-23

**Authors:** Joy Scaria, Aswathy Sreedharan, Yung-Fu Chang

**Affiliations:** 1 Department of Population Medicine and Diagnostic Sciences, Cornell University, Ithaca, New York 14853, USA; 2 Department of Plant Pathology, Cornell University, Ithaca, New York 14853, USA

## Abstract

**Background:**

Microarrays are becoming a very popular tool for microbial detection and diagnostics. Although these diagnostic arrays are much simpler when compared to the traditional transcriptome arrays, due to the high throughput nature of the arrays, the data analysis requirements still form a bottle neck for the widespread use of these diagnostic arrays. Hence we developed a new online data sharing and analysis environment customised for diagnostic arrays.

**Methods:**

Microbial Diagnostic Array Workstation (MDAW) is a database driven application designed in MS Access and front end designed in ASP.NET.

**Conclusion:**

MDAW is a new resource that is customised for the data analysis requirements for microbial diagnostic arrays.

## Background

Although microarrays were originally developed and mostly used for applications like gene expression profiling and Comparative Genome Hybridizations (CGH)[1], recently they are becoming more popular for diagnostic applications like microbial identification and detection[2,3]. The quantum jump in the number of publications in various journals describing development of such microbial diagnostic arrays is an indication of this trend. Such adoptions are made possible by the plummeting cost of array, the availability of large number of sequenced microbial genomes and access to array design programs like E-array . In contrast to two color microarrays used in transcriptome analysis, diagnostic arrays typically are labelled in single color and have only far less number of probes there by reducing the data analysis complexity. Based on the fluorescence intensity, probes are usually classified as present or absent. Subsequently, this presence/absence pattern is converted manually into inventory list of microbes present in the analyzed samples [4]. Yet, performing these steps manually becomes cumbersome with increasing number of probes per array and a large sample set. Even though several groups are developing such diagnostic arrays, as pointed out in the review by Loy and Bodrossy [4], lack of an easy to use software constitutes a major bottleneck of array based diagnostics. Hence we have developed a web server called Microbial Diagnostic Array Workstation (MDAW), specifically for diagnostic array data sharing and analysis.

## Methods

**Figure 1:**
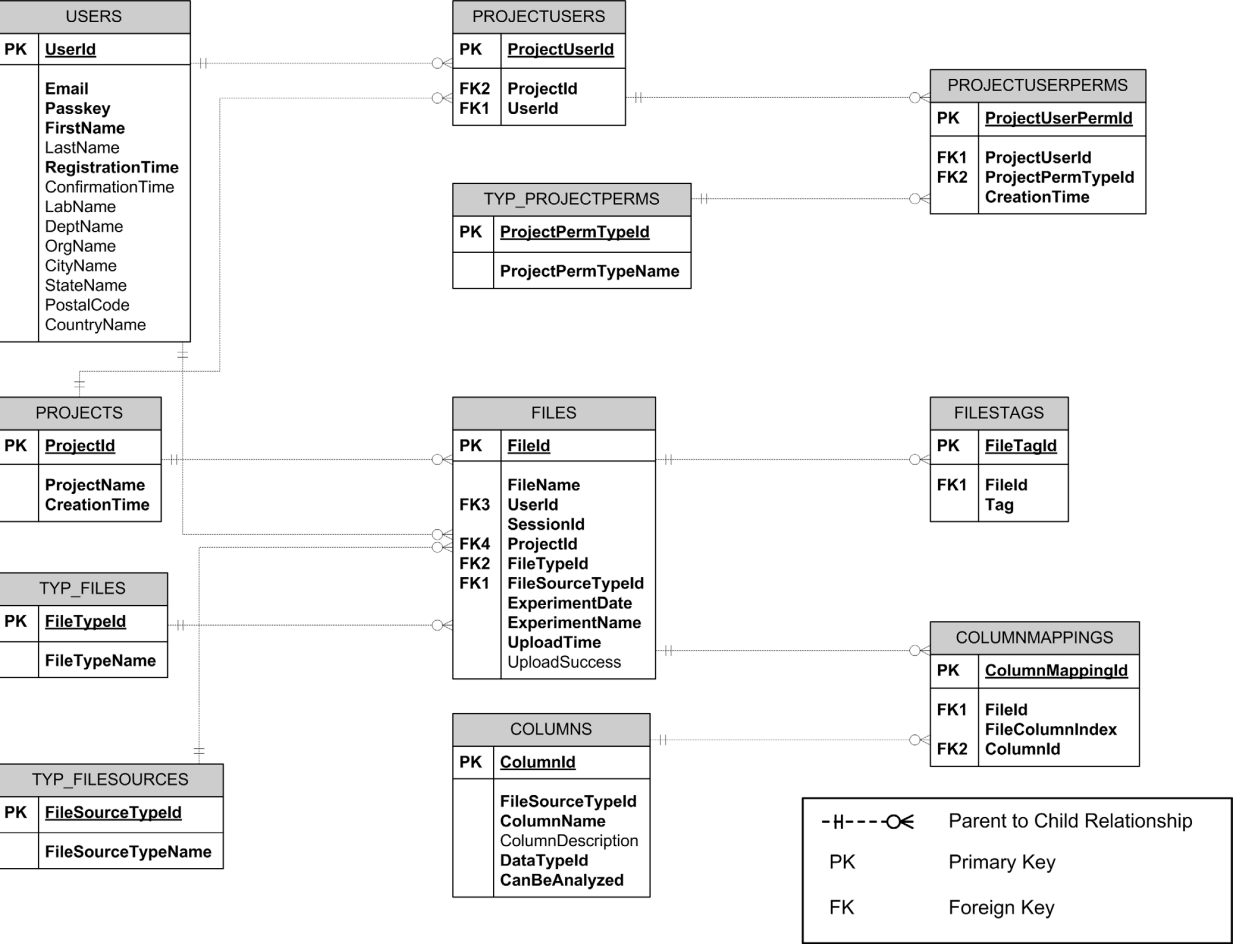
**Database schema implemented in MDAW**. User management, data storage and analysis is distributed as 11 tables. For a local implementation of the database these tables can created with the standard MS Access table and query wizards.

MDAW is a database driven application with the back end designed in Microsoft Access. The data parsing and analysis scripts are written in ASP.NET. The web interface is developed in ASP.NET and runs on an Apache web server and can be freely accessed the domain . The default input data format is gene pix pro (gpr). If the data is in any other format then it needs to be converted to tab delimited text (.txt) before uploading. The annotation file which is optional need to be in comma separated value (cvs) format. Figure. 1 explains further details of the implementation.

## Results

### Server access, user management and privileges

Considering the fact that often diagnostic array projects are collaborative in nature and the requirements of sharing the raw data or results with multiple users across several organizations or even countries, we made MDAW as an online resource. It has two modes of usage. The full analysis mode requires free user registration and will allow data storage, sharing, project collaboration and analysis. In this mode, all registered users are allowed to work in a password-protected environment, private from all other users. Also users get 1 GB of space for data and results storage. Users can upload any number of files within this storage limit. For better file management, files can be grouped into different projects, experiments, dates and for searching and indexing one or more tags can be added to each file. For collaborative projects, users also can designate themselves as principal investigators and add other users to the project space. Options for specifying limited or full access rights are available. The second mode named "Ad-hoc analysis" does not require any registration and have all options in the full user mode except for data storage and sharing. This mode can be accessed from the main page of MDAW by going to the link "Run ad-hoc analysis" . This is suggested mode of access for those users who does not require any online data storage or sharing but just utilize the data analysis capabilities of MDAW. As the name Ad-hoc analysis indicates, in this mode users can not store any data in the server and the results need to exported to local machine or will be lost upon closing the browser.

### Data management

All the files in MDAW is arranged under a project. Hence before uploading the raw data files, user need to create at least one project. The default data format for MDAW is Genepix Pro format (gpr). If the data is in any other format it needs to be transformed into a tab delimited text file. For facilitating file management, user also will be prompted to add the date of file uploading, experiment name and one or more tags for future searches. While uploading Genepix format files, MDAW server automatically detects the labeling wavelength (Cy3 or Cy5) and displays the data columns it is going to use for subsequent normalization and analysis. In this process of data column mapping, user have the option of accepting the automatically detected mapping pattern or change the mapping manually. If a tab delimited file is uploaded, all the column headers will be displayed and user will be asked to map the displayed headers to the required column headers. All the files can be then searched based on projects, experiments, date and tags. Downloading the files or deleting them also is possible from the same page.

### Data merging and normalization

**Figure 2:**
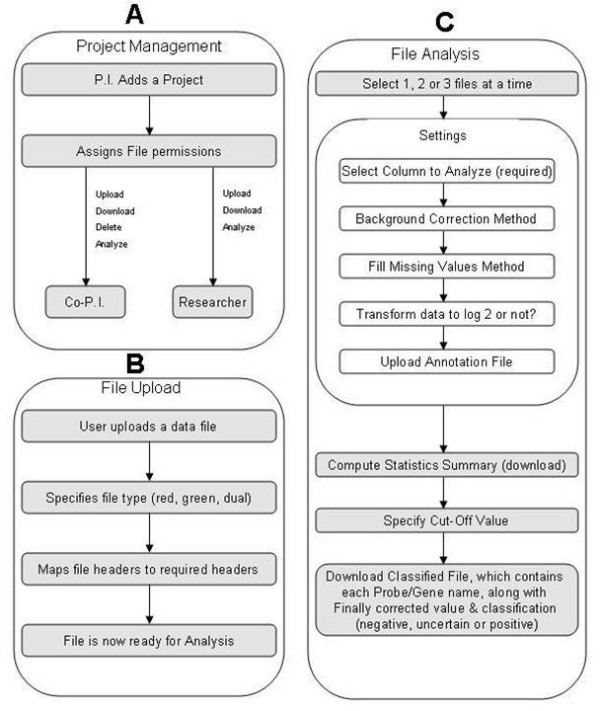
**File management and data analysis in MDAW**. Panel A; Project management has three levels if rights. The Principal investigator (PI) has full access rights to the projects space and files. PI can add one or more Co-PI and Researchers. Depending on the access rights granted by PI, Co-PI and Researchers will have full or limited access to the project space and files. Panel B; File upload require user to specify the type of files and column headers for analysis. If the file is in the default GPR format, the column headers and signal type is automatically detected. Panel C; The analysis pipeline implemented in MDAW. The directed workflow allows user to select all options or skip one or more of them.

The analysis pipeline (Figure. 2) in MDAW accommodates most of the analysis methods described in diagnostic array literature. It adopts a directed workflow, yet is very flexible and users have the freedom to accept or skip one or more steps. When compared to CGH and expression profiling arrays, diagnostic microarrays follow different but simple normalization and analysis workflow. In most instances, the type of signal analyzed is the mean/median signal intensity[2,5] or the Signal to Noise Ratio (SNR) [6-8]. Then the mean of the replicates are taken and background fluorescence intensities may be subtracted [3,9]. The missing probe intensities then could be filled in and the values are log transformed [10]. All these have been simplified in MDAW and users can perform all the above by clicking on the numbered radio buttons given in analysis page.

### Annotation file

The annotation file for a microarray is a file usually containing details like the probe sequence, the name of the gene for which the probe is designed and further information aiding with data analysis. The final step before the calculation of summary statistics in MDAW is uploading an annotation file for the array. Although optional, in addition to combining the results with the probe annotation, the annotation file which need to be in comma separated value format (csv) has a number of very important utilities. Mostly the probes in the array will be printed in random and the resultant data file will have the probe results arranged in random. In conventional array analysis, the program gives a set of genes that are up regulated or down regulated and the order in which the probes appear does not matter. However the diagnostic arrays pose a different scenario. Here mostly the probes are classified based on a set of unchanging positive controls and based on these probes values, other probes are classified as present or absent[2]. In MDAW, user can upload an annotation file containing the positive controls or any set of probes of choice arranged in a particular sort order, and then the server will calculate the summary statistics for only the probes in annotation file. When the results are exported, that will contain only those probes that were present in the annotation file and in the same order of the probes in the annotation file. Thus this is a very handy tool to extract the information for a subset of probes from the array or to calculate the results based only on the positive controls, common genes or any other statistical control included. By making different sets of annotation files, users can export probes in the order they want or in any combination they like.

### Calculation of summary statistics and probe classification

Once the replicates are merged, background correction, log transformation and adding annotation file is performed, summary statistics of the probes is carried out. Based on this the users can then select the method of probe classification. The most common methods of probe classification in diagnostic arrays can be grouped into three categories. In the first approach, the probes are classified as positive and negative based on the values of a set of control probes. The parameter could be mean/median of the selected probe set or standard deviation [2,11,12]. In the second method, the probes are classified based on a fixed cut off value[5,13]. Based on a control set the third method classifies the probes as positive, negative and uncertain. This method of introducing a window of uncertain category of probes reduces the false positives and negatives [3]. Almost all of the diagnostic array literature uses one of the above methods or a slight variant of these. In MDAW we have implemented all these three types and user can select any one of this method from a simple pull down menu from the final result export page. Once the method is selected, the results are exported as a CSV file and can be opened in any popular program like Microsoft Excel. Detailed explanation of each step is given in the help section of the MDAW. Also this section contains several video tutorials explaining how to use MDAW.

## Conclusion

There are a number of online and standalone programs for the analysis of different types of microarray data [14-18]. Although all of these have several normalization and clustering methods, adapting these programs for diagnostic arrays will require many roundabout steps or combining one or more different programs. Also the current online microarray data analysis programs require the users to submit the analysis requests and wait long time or get the results emailed later. In contrast to this, MDAW offers a very flexible directed workflow for diagnostic arrays and offer instant analysis results. There for it combines the speed of stand alone programs and the convenience of access from any internet connected computer.

## Competing interests

The authors declare that they have no competing interests.

## Availability and requirements



The full analysis mode of MDAW which require free registration is available at: 



The ad-hoc analysis mode which does not need any registration is available freely at:



## Authors' contributions

JS conceived the work and designed the analysis pipeline and help section. AS developed the database backend and GUI. YFC was involved in the supervision and preparation of the manuscript. All authors read and approved the final manuscript.
